# Molecular alterations of isocitrate dehydrogenase 1 and 2 (*IDH1 *and *IDH2*) metabolic genes and additional genetic mutations in newly diagnosed acute myeloid leukemia patients

**DOI:** 10.1186/1756-8722-5-5

**Published:** 2012-03-07

**Authors:** Sadudee Chotirat, Wanna Thongnoppakhun, Orathai Promsuwicha, Chetsada Boonthimat, Chirayu U Auewarakul

**Affiliations:** 1 Department of Immunology, Mahidol University, Bangkok, Thailand; 2 Department of Research and Development, Mahidol University, Bangkok, Thailand; 3 Department of Medicine, Faculty of Medicine Siriraj Hospital, Mahidol University, Bangkok, Thailand; 4 Division of Hematology, Department of Medicine, Faculty of Medicine Siriraj Hospital, Mahidol University, 2 Prannok Road, Bangkoknoi, Bangkok 10700, Thailand

**Keywords:** Acute myeloid leukemia, Isocitrate dehydrogenase, Metabolic enzymes, *IDH1*, *IDH2*, Cooperative mutations, Normal karyotype

## Abstract

**Background:**

Isocitrate dehydrogenase 1 and 2 (*IDH1 *and *IDH2*) metabolic genes encode cytosolic and mitochondrial enzymes that catalyze the conversion of isocitrate to α-ketoglutarate. Acquired somatic mutations of *IDH1 *and *IDH2 *have recently been reported in some types of brain tumors and a small proportion of acute myeloid leukemia (AML) cases.

**Methods:**

Two-hundred and thirty newly diagnosed AML patients were analyzed for the presence of *IDH1 *and *IDH2 *heterozygous mutations by polymerase chain reaction-denaturing high performance liquid chromatography (PCR-DHPLC) followed by direct sequencing. Clinical and biological characteristics were analyzed and correlated to the *IDH *mutational status. Coexisting mutations such as *FLT3*, *PML-*RARA, *RAS*, *AML1*, and *NPM1 *mutations were additionally explored.

**Results:**

The prevalence of *IDH1 *and *IDH2 *mutations was 8.7% (20/230) and 10.4% (24/230), respectively. Six missense mutations were identified among *IDH1*-mutated cases; p.R132H (n = 8), p.R132C (n = 6), p.R132S (n = 2), p.R132G (n = 2), p.R132L (n = 1), and p.I99M (n = 1). Two missense mutations were found in *IDH2*-mutated cases; p.R140Q (n = 20) and p.R172K (n = 4). No patients had dual *IDH1 *and *IDH2 *mutations. About 18% of AML with normal cytogenetics and 31% of acute promyelocytic leukemia had *IDH *mutations. Half of the *IDH*-mutated cohort had normal karyotype and the major FAB subtype was AML-M2. Interestingly, *IDH1*- and *IDH2*-mutated cases predominantly had *NPM1 *mutations (60-74%) as compared to the wild type (P < 0.001). Very few *IDH*-mutated cases had *FLT3 *and/or *RAS *abnormalities and none of them had *AML1 *mutations. Older age and higher median platelet counts were significantly associated with *IDH2 *mutations although the clinical impact of either *IDH1 *or *IDH2 *mutations on patients' overall survival could not be observed.

**Conclusion:**

Overall, 19% of newly diagnosed AML patients had alterations of *IDH *genes. No patients concurrently carried both *IDH1 *and *IDH2 *mutations suggesting that these mutations were mutually exclusive. *NPM1 *mutation appears as a major coexisting genetic mutation in *IDH*-mutated patients. Our present data failed to support the prognostic relevance of *IDH *mutations although alterations of these metabolic genes potentially have an important role in leukemia development.

## Background

Acute myeloid leukemia (AML) is a malignant hematologic disorder characterized by abnormal expansion of differentiation-defective myeloid cells [1]. Various chromosomal aberrations have been identified in AML patients and are uniquely associated with distinct clinical entities and prognostic relevance [2]. Although 40-50% of AML cases do not carry any detectable chromosomal abnormalities, a fraction of them are found to have mutations of genes that normally function in cell proliferation, differentiation, and survival such as *FLT3*, *NPM1*, *RAS*, *WT1*, and *AML1 *[3,4]. Moreover, through a rapid whole genome sequencing approach, it is now evident that at least half of the AML cases with normal karyotype have readily identifiable genomic abnormalities [5].

Alteration of cellular metabolism has recently been proposed as a novel oncogenetic mechanism [6,7]. Isocitrate dehydrogenase (IDH) is one the enzymes that, if defective, lead to abnormal cellular metabolism [8,9]. There are three IDH isoforms; IDH1 is in the cytoplasm whereas IDH2 and IDH3 are localized in the mitochondria [9,10]. *IDH1 *and *IDH2 *genes encode enzymes that catalyze oxidative decarboxylation of isocitrate into α-ketoglutarate (α-KG) by utilizing nicotinamide adenine dinucleotide (NAD) or NAD phosphate (NADP) as a cofactor to generate NADH or NADPH, respectively [11]. In 2008, a novel mutation of *IDH1 *gene was firstly described in patients with glioblastoma multiforme (GBM). Subsequent studies additionally identified such mutations in > 70% of young adults with low-grade glioma and 80% of patients with secondary GBM [12-14]. Meanwhile in 2009, *IDH1 *mutation was reported in a subset of AML patients lacking specific chromosomal aberrations [5] and in 2010, *IDH2 *mutation was identified in AML, myelodysplastic syndrome (MDS), and myeloproliferative neoplasms (MPN) [15-17]. The worldwide frequencies of *IDH1 *and *IDH2 *mutations in newly diagnosed AML patients range from 2% to 14% and 1% to 19%, respectively, with the mutations mostly restricted to codon R132 of *IDH1 *and codon R140 of *IDH2 *[5,15,18-32]. Biochemical and molecular analyses reveal that mutations at the evolutionarily conserved site of *IDH *lead to interruption of the normal ability of enzyme to bind substrates and subsequent acquisition of novel enzymatic activity resulting in a substantial increase of oncometabolite *R*(-) -2-hydroxyglutarate (2HG) through α-KG conversion [8,19,20]. The accumulation of elevated 2HG induces global DNA hypermethylation and interruption of hematopoietic differentiation [33,34].

In the present study, we aimed to characterize *IDH1 *and *IDH2 *mutations in newly diagnosed AML patients and investigate their correlations to other parameters such as clinical and hematologic characteristics, cytogenetics and additional genetic mutations.

## Methods

### Leukemia samples

Leukemic samples from 230 newly diagnosed AML cases were consecutively recruited into the study. Clinical and biological characteristics were collected including clinical history, complete blood counts, peripheral blood (PB) smear, bone marrow (BM) studies, flow cytometric immunophenotyping, and chromosome analysis. Mononuclear cells (MNC) were isolated from the leukemic samples by Ficoll-Hypaque density-gradient centrifugation and subsequently used for molecular analysis. Twenty consented normal individuals were used as controls. Patients were treated according to the standard AML regimen which included idarubicin and cytarabine induction therapy followed by high-dose cytarabine-based consolidation phase. This study was approved by the Ethical Committee for Human Research, Faculty of Medicine Siriraj Hospital, Mahidol University.

### Mutational analysis of *IDH1 *and *IDH2*

Genomic DNA was extracted using standard phenol-chloroform method or Gentra Puregene Blood Kit (Qiagen, Hidden, Germany) according to the manufacturer's protocol. DNA amplicons harbouring exon 4 of *IDH1 *and *IDH2 *were amplified by polymerase chain reaction (PCR) using the primer pair; IDHIf (5'-AGCTCTATATGCCATCACTGC-3'), IDH1r (5'-AACATGCAAAATCACATTATTGCC-3'), IDH2f(5'- AATTTTAGGACCCCCGTCTG-3'), and IDH2r (5'-CTGCAGAGACAAGAGGATGG-3') [13]. PCR reactions were performed in a total volume of 20 μL containing 50 ng of genomic DNA, PCR master mixture consisting of 1x Phusion^®^HF Buffer (F-520), 200 μM dNTPs, 0.5 μM of each primer, 0.02 U/μL Phusion^® ^DNA polymerase, and Milli-Q water. The PCR was carried out in a Perkins Elmer PCR2400 thermal cycler (Applied Biosystems, Foster City, CA) using the following steps: initial denaturation at 98°C for 30 seconds (sec), 35 cycles at 98°C for 10 sec, 60°C for 30 sec. and 72°C for 30 sec, and final extension at 72°C for 5 minutes (min). Both amplicons were screened for heterozygous mutations by denaturing high-performance liquid chromatography (DHPLC) on a WAVE 3500HT with DNASep^® ^HT cartridge technology (Transgenomic Inc, Omaha, NE, USA). The optimized condition and temperature were predicted by the Navigator™ software to determine chromatographic peak pattern. PCR crude sample was injected into DHPLC column and the optimal temperature for IDH1 was 58.5°C and IDH2 was 64°C. Each DHPLC chromatogram was compared to a wild-type reference. The sensitivity of our assay was determined by performing a dilution series containing a different percentage (%) of mutant and wild-type IDH concentrations. Abnormal DHPLC peaks could be clearly detected in 50%, 20%, 10%, 5%, and 3.33% dilutions. The mutational chromatograms were re-amplified in an independent PCR reaction and further subjected to direct sequencing. The sequences were compared to the wild-type *IDH1 *and *IDH2 *cDNA (GenBank Accession number, NM_005896.2 and NM_002168.2, respectively) [25].

### Analysis of additional molecular aberrations

Mutational analyses of *FLT3*, *PML-RARA, RAS*, *AML1*, and *NPM1 *were performed according to our previously described method [35-39]. Briefly, the DNA or RNA was extracted, then the genes of interest were amplified and detected by gel electrophoresis (*FLT3*), denaturing high performance liquid chromatography (DHPLC) (*NPM1*), single-strand conformational polymorphism (SSCP) (*RAS *and *AML1*). For *PML*-*RARA*, the cDNA was synthesized and reverse transcriptase-polymerase chain reaction (RT-PCR) performed.

### Statistical analysis

The relationship between *IDH *mutations and various patient characteristics such as age, Hb count, WBC count, platelet count, and percentages of blasts was determined by the student *t*-test, equal variances not assumed for continuous variables. Categorical variables such as FAB classification, cytogenetics, and test. The Kaplan-Meier method and the log-rank test were utilized to estimate the distribution of OS [40]. For all analyses, a p-value of less than 0.05 was considered statistically significant. All reported p-values were 2-sided.

## Results

### Frequency and type of *IDH1 *and *IDH2 *mutations

**Figure 1:**
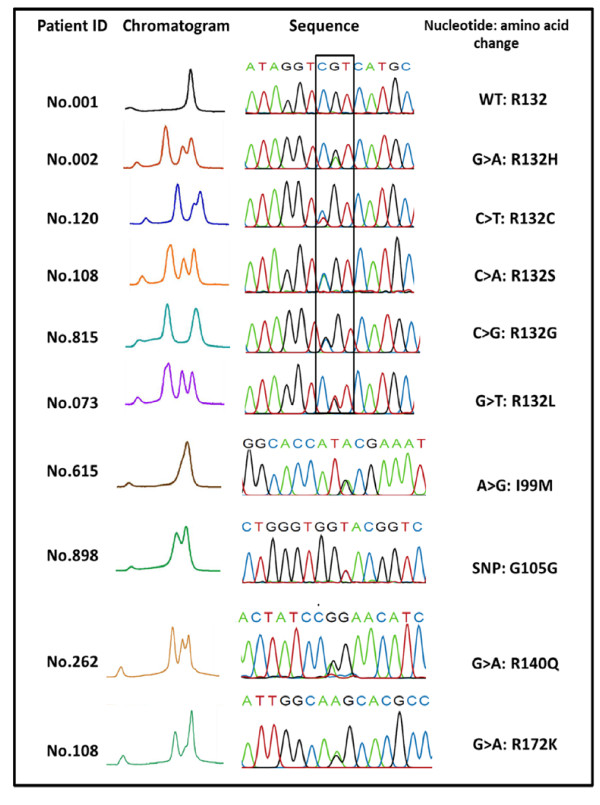
**Detection of *IDH1 *and *IDH2 *mutations by DHPLC and direct sequencing analysis**. DHPLC patterns shows a suspicious peak in cases with *IDH1 *or *IDH2 *mutations as compared to cases with the wild type (WT); Six missense *IDH1 *mutations; R132H, R132C, R132S, R132G, R132L, and I99M and 1 silent mutation (G105G), and 2 missense *IDH2 *mutations; R140Q and R172K, are demonstrated. (*Abbreviation*: C, cysteine; G, glycine; H, histidine; I, isoleucine; K, lysine; L, leucine; M, methionine; Q, glutamine; S, serine).

In a total cohort of 230 consecutive AML patients (36 acute promyelocytic leukemia (APL) and 194 non-APL), 44 patients with *IDH *mutation were identified (19.13%) by DHPLC showing abnormal chromatogram patterns that were different from the wild-type profiles. These mutations were further confirmed by sequencing analysis (Figure 1). Twenty *IDH1 *mutations (8.7%) included six missense mutations leading to amino acid (AA) substitution with different frequencies: c.G395A; p.R132H in 8 cases (40.0%), c.C394T; p.R132C in 6 cases (30.0%), c.C394A; p.R132S in 2 cases (10.0%), c.C394G; p.R132G in 2 cases (10.0%), c.G395T; p.R132L in 1 cases (5.0%), and c.A297G; p.I99M in 1 cases (5.0%) (Table 1). In addition, one silent polymorphism (c.315 G > T; *IDH1^G105G^*) was observed in 3 patients (1.30%). Twenty-four *IDH2 *mutations (10.4%) included two missense mutations with different frequencies: c.G419A; p.R140Q in 20 cases (83.3%) and c.G515A; p.R172K in 4 cases (16.7%). All patients with the mutations were heterozygous and retained a wild-type allele (Figure 1). No mutated patients harbored dual mutations of both genes, indicating that these mutations are mutually exclusive.

**Table 1:** Type of *IDH1 *and *IDH2 *mutations identified in 230 AML patients

Mutation	Nucleotide change	Predicted protein change	No. of patients
IDH1*			
c.G395A	CGT-CAT	p.R132H	8
c.C394T	CGT-TGT	p.R132C	6
c.C394A	CGT-AGT	p.R132S	2
c.C394G	CGT-GGT	p.R132G	2
c.G395T	CGT-CTT	p.R132L	1
c.A297G	ATA-ATG	p.I99M	1
IDH2**			
c.G419A	CGG-CAG	p.R140Q	20
c.G515A	AGG-AAG	p.R172K	4

*Abbreviation: *AML, acute myeloid leukemia; C, cysteine; G, glycine; H, histidine; I, isoleucine; K, lysine; L, leucine; M, methionine; Q, glutamine; S, serine

*IDH1 nucleotide numbering based upon the National Center for Biotechnology Information sequence NM_005896.2

**IDH2 nucleotide numbering based upon the National Center for Biotechnology Information sequence NM_002168.2

### Clinical parameters and morphologic subtypes of patients with *IDH1 *or *IDH2 *mutations

Twenty *IDH1*-mutated patients showed no significant differences in age, hemoglobin level, WBC counts, platelet counts or percentages of blasts as compared to the *IDH1 *wild-type group, although a trend towards more females was observed (15 females vs 5 males; *P *= .058). Twenty-four *IDH2-*mutated patients also showed no significant differences in sex, WBC counts or percentages of blasts but an older age (49.5-year vs. 43-year; *P *= .001), a higher platelet count (59 vs. 45 × 10^9^/L; *P *= .048), and a trend towards higher hemoglobin level (9.25 g/dL vs. 7.7 g/dL; *P *= .059) were observed in the *IDH2*-mutated group as compared to the wild-type group (Tables 2 and 3). Among *IDH1*-mutated cases, the majority was classified as AML with maturation (AML M2) (13/20 cases, 65%) followed by APL (5/20 cases, 25%). None of the cases with APL, acute monoblastic or monocytic leukemia, acute erythroid leukemia, and acute megakaryoblastic leukemia was found among *IDH1*-mutated group. Similarly, AML with maturation (AML M2) was the most common subtype among the *IDH2*-mutated group (11/24 cases, 46%) (Table 3). The second most common subtypes were APL (6/24 cases, 25%) and acute myelomonocytic leukemia (6/24 cases, 25%). The OS of patients with or without *IDH1 *or *IDH2 *mutations did not differ among the entire series of AML patients and AML patients with normal cytogenetics (*P *= 0.200 and 0.272, respectively). No significant difference was demonstrated in subgroups of younger patients (age < 60) and patients with/without additional mutations as compared to the wild-type patients (*P *= 0.471 and 0.812, respectively).

**Table 2:** Characteristics of AML patients with a wild type or mutated IDH1

Variable	All cases	*IDH1^m^*	*IDH1*^wt^	P*
**No. of cases**	230	20	210	
**No. of males/females**	101/129	5/15	96/114	.058
**Age, years**				.860^†^
Median (range)	44 (13-86)	42.5 (15-85)	45 (13-86)	
**Hemoglobin, g/dL**				.311^†^
Median (range)	7.8 (1.4 -14.5)	7.4 (2.9 - 10.8)	7.85(1.4-14.5)	
**WBC count, x10^9^/L**				.323^†^
Median (range)	27.7 (0.6 -494.4)	26.9 (1.0 -190.9)	27.7 (0.6 -494.4)	
**Platelet, x10^9^/L**				.923^†^
Median (range)	46 (1.8-965)	56 (16.9 - 386)	45.5 (1.8-965)	
**Percentage of blasts**				.738^†^
Median (range)	70.19 (6.69-95.44)	75.8 (13.3-91.4)	69.77 (6.69-95.44)	
		Patients (%)	Patients (%)	

**FAB classification**				
M0	3	1 (5)	2 (0.9)	
M1	51	1 (5)	50 (23.8)	
M2	76	13 (65)	63 (30)	
M3	36	5 (25)	31 (15)	
M4	34	0 (0)	34 (16)	
M5	21	0 (0)	21 (10)	
M6	8	0 (0)	8 (3.8)	
M7	1	0 (0)	1 (0.5)	
		Patients (%)	Patients (%)	

**Cytogenetics**				
Abnormal				
t(15;17)	36	5 (25)	31 (15)	.184
t(8;21)	20	0 (0)	20 (10)	.149
inv(16)	3	0 (0)	3 (1)	.760
trisomy 8	4	1 (5)	3 (1)	.307
Other trisomies	7	0 (0)	7 (3)	.524
All others including monosomies, deletions or combination of these	22	2 (10)	20 (10)	.594
Complex karyotype	8	0 (0)	8 (4)	.477
Normal	126	11 (55)	115 (55)	.587

Abbreviation: IDH1^m^, IDH1 mutated; IDH1^wt^, IDH1 wild type

* Fisher exact test unless otherwise indicated; ^†^, the student *t*- test

**Table 3:** Characteristics of AML patients with a wild type or mutated *IDH2*

Variable	All cases	*IDH2*^m^	*IDH2*^wt^	P*
**No.of cases**	230	24	206	
**No.of males/females**	101/129	11/13	90/116	.504
**Age, years**				**.001**^†^
Median (range)	44 (13-86)	49.5 (38-80)	43 (13-86)	
**Hemoglobin, g/dL**				.059^†^
Median (range)	7.8 (1.4 - 14.5)	9.25 (4.7-12.6)	7.7 (1.4 - 14.5)	
**WBC count, × 10^9^/L**				.789^†^
Median (range)	27.7 (0.6 - 494.4)	29.73 (0.89-222)	26.4 (0.6 - 494.4)	
**Platelet, x10^9^/L**				**.048^†^**
Median (range)	46 (1.8-965)	59 (11-965)	45 (1.8-852)	
**Percentage of blasts**				.133 ^†^
Median (range)	70.19 (6.69-95.44)	61.79 (32.52-95.03)	71.66 (6.69-95.44)	
		Patients (%)	Patients (%)	

**FAB classification**				
M0	3	0 (0)	3 (1.5)	
M1	51	1 (4)	50 (24)	
M2	76	11 (46)	65 (31)	
M3	36	6 (25)	30 (15)	
M4	34	6 (25)	28 (14)	
M5	21	0 (0)	20 (10)	
M6	8	0 (0)	8 (4)	
M7	1	0 (0)	1 (0.5)	
		Patients (%)	Patients (%)	

**Cytogenetics**				
Abnormal				
t(15;17)	36	6 (25)	30 (14.6)	.150
t(8;21)	20	1 (4.2)	19 (9.2)	.355
inv(16)	3	0 (0)	3 (1.5)	.717
trisomy 8	4	1 (4.2)	3 (1.5)	.358
Other trisomies	7	2^‡ ^(8.2)	5 (2.4)	.158
All others including monosomies, deletions or combination of these	22	1 (4.2)	21 (10.2)	.301
Complex karyotype	8	0 (0)	8 (3.8)	.408
Normal	126	12 (50)	114 (55.3)	.388

Abbreviation: IDH2^m^, IDH2 mutated; IDH2^wt^, IDH2 wild type

* Fisher exact test unless otherwise indicated; ^†^, Student t- test; ^‡^, 2 cases of trisomy11 harboring IDH2 R172K mutation

### Chromosomal patterns and additional molecular aberrations in patients with *IDH1 *or *IDH2 *mutations

Cytogenetic information was available in 226 of 230 patients, 126 patients (55.75%) of whom had normal karyotype and 100 patients (44.25%) had an aberrant karyotype. Of 20 AML cases with *IDH1 *mutation, 11 cases had normal karyotype (55%). In the aberrant karyotype, we found 5 cases with t(15;17) chromosome translocation, 2 cases with del(9q), and 1 case with trisomy 8 (Table 2). Of 24 cases with *IDH2 *mutation, 12 cases had normal karyotype (50%). In the aberrant karyotype, we observed 6 cases with t(15;17), 2 cases were *IDH2 *R172K harboring trisomy 11, 1 case with trisomy 8, 1 case with t(8;21), and 1 case with del(12)(p12.1p13.1) (Table 3).

*IDH1 *mutations were significantly associated with *NPM1 *mutation as compared with wild-type cases (14/19, 74% vs. 45/185, 24%; *P *< 0.001)(Table 4). Some cases had additional mutations including *FLT3*-ITD, *FLT3*-TKD, *NRAS, and PML-RARA*. *IDH2 *mutations were also significantly associated with *NPM1 *mutations when compared with the respective wild-type cases (12/20, 60% vs. 47/184, 26%; *P *< 0.001)(Table 4). *AML1 *mutations were not observed in any mutated *IDH1 *or *IDH2 *patients.

**Table 4:** Comparison of additional gene mutations in AML patients with and without *IDH1 *and *IDH2 *mutations

Additional gene mutations	*IDH1^m^* **n = 20 No**.	(%)	***IDH1^wt ^*n = 210 No**.	%	P*	***IDH2^m ^*n = 24 No**.	%	***IDH2^wt ^*n = 206 No**.	%	P*
***NPM1***					**<.001**					**<.001**
Wild type	5	26%	140	76%		8	40%	137	75%	
Mutated	14	74%	45	24%		12	60%	47	25%	
***FLT3-*ITD**					.34					.63
Absent	14	82%	108	74%		13	76%	109	75%	
Present	3	18%	38	16%		4	24%	37	25%	
***FLT3-*TKD**					.59					.59
Absent	15	94%	133	91%		15	94%	133	91%	
Present	1	6%	33	9%		1	6%	33	9%	
***AML1***					.09					.09
Wild type	15	100%	147	86%		17	100%	145	86%	
Mutated	0	0%	23	14%		0	0%	23	4%	
***NRAS***					.49					.42
Wild type	8	89%	79	81%		9	90%	78	81%	
Mutated	1	11%	18	19%		1	10%	18	19%	
***PML*-RARA**					.43					.56
Wild type	3	38%	12	28%		2	25%	13	30%	
Mutated	5	62%	31	72%		6	75%	30	70%	

## Discussion

In the present study, we developed a screening DHPLC method followed by sequencing analysis to detect and confirm the presence of *IDH *mutations in newly diagnosed AML patients. Twenty cases of *IDH1 *mutations and 24 cases of *IDH2 *mutations were discovered among the entire newly diagnosed AML cohort. Previous reports from the Asia continent were available from two countries, i.e. Taiwan [18] and China [28,31,32] while the Western studies were from USA [5,15,20,22,29], Canada [19], France [41], Germany [21,23,25,27], the Netherlands [24], and UK [26,30] (Table 5). The overall frequency of *IDH *mutations appears to vary between 2-14% for *IDH1 *and 1-19% for *IDH2 *from most Western reports [5,15,19,27,29,30,41]. Worthy of note, the frequency of *IDH1 *mutations in our population of 8.4% was comparable to 8.5% in the first study reported by Mardis *et al. *in 2009 [5] although these figures were somewhat higher than those of the Chinese AML studies (5.5%, 5.6%, 6.3%, and 3.6%) [18,28,31,32]. The frequency of *IDH2 *mutations of 10.4% in our cases was also slightly higher than the only available *IDH2 *study from Asia (8.3%, 4/48) [28]. The frequency discrepancies among various studies may reflect the variable inclusion criteria of the study samples, the variable sensitivity of the detection assays, the selective inclusion or exclusion of certain *IDH *aberrations or the true racial differences.

**Table 5:** Incidence of *IDH *mutations in AML patients from various countries

Country	**No**. ***IDH1m*/all cases (%)**	No. of *IDH1m*/CN-AML (%)	**No**. ***IDH2m*/all cases (%)**	No. of *IDH2m*/CN-AML (%)
**ASIA**				
**Taiwan**				
Chou W *et al.*(2010) [18]	27/493 (5.5)	20/227 (8.8)	ND	ND
**China**				
Zou Y *et al.*(2010) [28]	4/68 (5.9)	ND	4/48 (8.3)	ND
Zhang Y *et al.*(2011) [32]	23/365 (6.3)	6/111 (5.4)	ND	ND
Lin J *et al.*(2011) [31]	4/110 (3.6)	ND	ND	ND
**Thailand**				
This study	20/230 (8.7)	11/126 (8.7)	24/230 (10.4)	12/126 (9.5)
**EUROPE**^‡ ^[21,23-27,30,41]	6.0-10.9	9.4-16.0	2.0*-10.9	3.2*-15.2
**NORTH AMERICA^∫^**	2.2-13.7	4.9-16.0	1.3*-19.2	2.4*-19.2
[5,15,19,20,22,29]				

*The authors only identified *IDH2 *R172K but did not include *IDH2 *R140Q mutation; ^† ^Pediatric acute myeloid leukemia; ^‡ ^All studies reported during 2010-2011; ^∫ ^All studies reported during 2009-2011; ND, not determined

*IDH1 *mutations consisting of six different amino acid exchanges at p.R132 (n = 19) and p.I99M (n = 1) were identified. Within the p.R132 group, arginine was replaced by histidine (R132H) in most cases (n = 8, 40%), followed by cysteine (R132C; n = 6, 30%), serine (R132S; n = 2, 10%), glycine (R132G; n = 2, 10%) and leucine (R132L, n = 1, 5%). This pattern was extremely different to the mutation pattern reported in glioma, where R132H was predominant observed in 88% of all cases while R132C present in only 4.5% [13]. To date, results from structural and functional assays by several multicenter trials suggested that *IDH1 *R132, which resides at the active site of enzyme substrate affinity, promotes oncogenesis in both glioma and AML [9,11,20,33]. In the p.I99M case, isoleucine was substituted by methionine which was recently identified as a novel missense mutation in the Chinese cohort by Zou *et al *[28]. The same study revealed that this evolutionary point mutation was also located in the substrate binding site of enzyme and may drive pathogenesis; however, the exact mechanism needs further investigation. In addition, we detected one silent polymorphism (*IDH1*^G105G^) in 3 cases (1.3%). Wagner *et al. *[21] previously reported that *IDH1*^G105G ^allele conferred an adverse prognostic impact to patients' survival.

The identified *IDH2 *mutations involved two different types of amino acid substitution spanning exon 4 of the *IDH2 *gene at arginine 140 and arginine 172. Of note, the former arginine was replaced by glutamine (R140Q; n = 20, 83.3%) and the latter arginine was replaced by lysine (R172K; n = 4, 16.7%). Our study was similar to previous studies which revealed that more than 80% of the *IDH2 *mutations involved R140 [15]. R172 mutations were profoundly associated with biological insights and clinical outcome [15,20] while R140 has not been addressed to associate with any prognostic significance in AML [23]. Therefore, functional validation should be employed to define whether R140 plays a significant role in AML pathogenesis or is simply a genuine polymorphism.

*IDH1 *mutation was previously reported to be strongly associated with normal karyotype or intermediate risk karyotype AML [5,15,25,41]. Noticeably, our present study found that although *IDH1 *mutation predominantly had normal karyotype (n = 11/20), various aberrant karyotype were also found (n = 8/20) including 5 cases of t(15;17). Similarly, although half of *IDH2*-mutated cases had normal karyotype (n = 12/24), 6 cases had t(15;17). Our study showed a higher frequency of *IDH *mutations in APL with t(15;17) (n = 11/36 cases, 31%) than most other APL series reported [5,18,24,27,29,32] (Table 6). The prognostic significance of *IDH *mutations in APL patients needs further studies.

**Table 6:** Reported frequencies of *IDH1 *and *IDH2 *mutations in APL patients worldwide

Country	**No**. ***IDH1m*/all cases (%)**	No. of *IDH1m *in APL (%)	**No**. ***IDH2m*/all cases (%)**	No. of *IDH2m *in APL (%)
**ASIA**				
**Taiwan**				
Chou W *et al.*[18]	27/493 (5.5)	0/37 (0)	ND	ND
**China**				
Zhang Y *et al.*[28]	23/365 (6.3)	3/77 (3.9)	ND	ND
**Thailand**				
This study	20/230 (8.7)	5/36(13.9)	24/230 (10.4)	6/36 (16.7)
**EUROPE**				
**Germany**				
Schnittger S *et al.*[27]	93/1414 (6.6)	2/88 (2.3)	ND	ND
**The Netherlands**				
Abbas S *et al.*[24]	55/893 (6.2)	0/21 (0)	97/893 (10.9)	0/21 (0)
**NORTH AMERICA**				
**United States**				
Mardis E *et al.*[5]	16/188 (8.5)	0/16 (0)	ND	ND
Andersson A *et al.*[29]	5/227 (2.2)	0/7 (0)	3/227 (1.3)	0/7 (0)

ND, not determined

To explore if other genetic mutations coexist in AML cases with *IDH *mutations, we performed mutation analysis of various different genes, i.e. *FLT3*, *NPM1*, *NRAS *and *AML1*. *IDH1 *mutations were found to be most frequently accompanied by *NPM1 *mutations (74% of the cases; *P *< 0.001). Previous studies also demonstrated that *IDH1 *mutation was significantly associated with *NPM1 *mutation, ranging from 12.5% to 67% as compared to the wild-type *IDH1 *cases [5,18,24,25,27,41]. Similarly, *IDH2 *mutations were significantly associated with *NPM1 *mutations (60% of the cases; *P *< 0.001) which were comparable to other reports [24-26]. No significant association was found with other molecular alterations including *FLT3*-ITD, *FLT3*-TKD, *NRAS *and *AML1 *although *FLT3*-ITD was also frequently found co-existing with *IDH1 *mutation in some studies [15,25]. Meanwhile, other authors also showed no significant correlation between either *IDH1 *or *IDH2 *mutation and *FLT3*-TKD, *NRAS *and *AML1 *mutation [5,18,24,27,30,41].

With respect to clinical and hematologic parameters, *IDH1 *mutated cases were frequently females rather than males (15 cases vs. 5 cases) which was similar to the German study by Schnittger *et al. *[27]. Interestingly, we observed that both *IDH1 *and *IDH2 *mutations were predominantly found in AML with maturation (AML-M2; n = 24/44) and acute promyelocytic leukemia (APL) (AML-M3; n = 11/44) which were different from AML-M1 as reported by others [5,27,30]. Interestingly, the frequency of *IDH2 *mutation coexisting in AML-M4 of 25% in our study was comparable with 27% in the finding reported by Thol *et al. *[23]. *IDH1 *or *IDH2 *mutations did not significantly impact survivals when the whole AML cohort or AML with normal karyotype analyzed (*P *= 0.200 and 0.272). We therefore further analyzed OS according to age and *NPM1 *status. Unfortunately, we could not find a significant difference between *IDH1*- and *IDH2*-mutated and wild-type cases (*P *= 0.471 and .812) either in the younger age group (< 60 years) or the *NPM1*-mutated genotype. Our study was consistent with some studies that revealed no impact of *IDH *mutations on the OS of AML cases although other studies suggested that *IDH1 *or *IDH2 *mutations conferred an adverse effect among AML with normal karyotype or AML with favorable genotype (*NPM1 *mutated/*FLT3 *wild type) [15,25-27,41]. *IDH1 *mutation conferred a shorter disease-free survival and *IDH2 *R172 mutation contributed to a lower complete remission or a higher relapse risk compared to wild-type *IDH *patients [25,41]. Our study may be limited by a small number of cases with *IDH *alterations and a substantial recruitment of cases with aberrant karyotype [18,23].

The possible oncogenic role of *IDH *mutations that contribute to AML development has been postulated by available evidence [20,33,34]. By structural and functional analysis, *IDH1 *and *IDH2 *mutated cells gained the neomorphic enzymatic activity creating a condition with 2HG oncometabolite accumulation which promotes tumorigenesis through inhibiting a cancer-associated transcription factor such as hypoxia-induced factor (HIF) [19,20,34]. Moreover, inhibition of normal myeloid differentiation and induction of global DNA hypermethylation by mutated *IDH *potentially lead to leukemogenesis [33], suggesting that *IDH *genes and their altered enzymatic pathways may be a potential new target for future drug development for AML patients. Intriguingly, *IDH1 *and *IDH2 *mutations were also found in other myeloid disorders such as myeloproliferative neoplasms (MPN) and myelodysplastic syndrome (MDS) which have a propensity to AML development, although at a much lower frequency than AML [42,43]. It was thus speculated that *IDH *mutations were likely to be associated with disease transformation or progression rather than disease initiation [44-46].

In conclusion, *IDH1 *and *IDH2 *mutations occur in a minor subset of newly diagnosed AML patients with a strong association with normal karyotype, AML-M2 subtype, and *NPM1 *mutation. No significant correlation with other mutations such as *FLT3*, *RAS*, and *AML1 *could be demonstrated. Larger studies are needed to confirm the prognostic impact of *IDH1 *and *IDH2 *mutations in AML patients from various ethnic backgrounds. Our results, nevertheless, provide a relevant rationale to utilize these genomic alterations to better characterize AML patients in the future.

## Competing interests

The authors declare that they have no competing interests.

## Authors' contributions

SC performed the experiments and data analysis and contributed to the drafting of the manuscript. WT supervised the molecular and data analysis and contributed to the revision of the manuscript. OP and CB contributed to *PML*-*RARA *and *NPM1 *mutational analyses. CUA was responsible for the initiation and execution of the entire project and critical revision of the manuscript. All authors read and approved the final manuscript.
